# Impact of a pharmaceutical care programme on health-related quality of life among women with epilepsy: a randomised controlled trial (IPHIWWE study)

**DOI:** 10.1186/s12955-014-0162-8

**Published:** 2014-10-31

**Authors:** Martha Losada-Camacho, Mario F Guerrero-Pabon, Pilar Garcia-Delgado, Fernando Martínez-Martinez

**Affiliations:** Pharmacy Department, Faculty of Sciences, National University of Colombia, Bogota, Colombia; Research Group FARMOL, National University of Colombia, Bogota, Colombia; Research Group CTS-131 Pharmaceutical Care University of Granada, Granada, Spain

**Keywords:** Epilepsy, Patient-reported outcomes (PROs), Pharmaceutical care, Quality of life, Randomised controlled trial, Women with epilepsy, Atención farmacéutica, Calidad de vida, Ensayo clínico controlado, Epilepsia, Mujer con epilepsia, Resultados reportados por los pacientes

## Abstract

**Background:**

Epilepsy is a complex chronic disorder which affects health-related quality of life (HRQOL), especially in women.

Pharmaceutical care (PC) allows direct intervention between the pharmacist, the patient and the other healthcare team members to optimise treatments in order to reduce negative outcomes related to medication and contribute to improving HRQOL.

The aim of the study was to establish the impact of the application of a pharmaceutical care programme on the HRQOL of women with epilepsy.

**Methods:**

This study is a pragmatic randomised controlled trial involving women with epilepsy (WWE) over 18 years of age.

The intervention group (IG) received a pharmaceutical care programme consisting of medication review follow-up according to Dáder’s method, health education and therapeutic drug monitoring of anticonvulsants.

The impact was assessed by changes in seizure frequency, in the self-administered questionnaires (the QOLIE-31, Liverpool AEP, CES-D, Haynes-Sackett test and Moriski-Green test) and between the first interview and the one at the end of six months of follow-up.

A Student’s t-test was performed to compare the final QOLIE-31 score between groups and a paired Student’s t-test was used to determine the change in each group between the start and the end of follow-up.

**Results:**

One hundred eighty-two WWE entered the study and 144 (79.1%) completed it. The t-test for comparing the final QOLIE-31 scores between groups yielded a t = −2.166 and confidence interval (CI) (95%): −10.125; −0.4625, p-value =0.0319. The change (Δ) in the QOLIE-31 score for the IG was 12.45 points (p-value <0.001) and for the control group it was 2.61 (p-value =0.072). With 10.7 as the minimally important change we found a relative risk of 2.17 (CI: 1.37; 3.43) and a number needed to treat (NNT) of 3.5.

**Conclusions:**

The study demonstrated that the application of a pharmaceutical care programme significantly improves HRQOL in WWE. The NNT we found allows a recommendation to implement the PC programme for the additional benefit that would be obtained in patients’ HRQOL.

**Trial registration:**

Current Controlled Trials ISRCTN46864306 IPHIWWE study.

## Resumen

**Antecedentes:**

La epilepsia es un desorden crónico complejo que afecta la calidad de vida relacionada con la salud (CVRS), especialmente en las mujeres.

La atención farmacéutica (AF) permite la interacción directa del farmacéutico con el paciente y con los demás integrantes del equipo de salud para optimizar los tratamientos con el fin de disminuir los resultados negativos asociados con la medicación y contribuir a mejorar la CVRS.

El objetivo del estudio fue establecer el impacto de la aplicación de un programa de AF en la CVRS de mujeres con epilepsia (MCE).

**Métodos:**

Ensayo clínico controlado aleatorizado pragmático en MCE mayores de 18 años.

Al grupo intervención se le aplicó un programa de AF consistente en seguimiento farmacoterapéutico según el método Dáder, educación para la salud y monitoreo de anticonvulsivantes.

El impacto se evaluó con el cambio en la frecuencia de crisis y de los cuestionarios auto-administrados (QOLIE-31, Liverpool AEP, CES-D, test de Haynes-Sackett, y test de Moriski-Green) entre la primera entrevista y al finalizar los seis meses de seguimiento.

Se realizó una *t de student* para comparar el puntaje final entre los grupos y una *t de student* pareada para comparar el cambio en el QOLIE-31 entre el inicio y el final en cada grupo.

**Resultados:**

Se incluyeron 182 pacientes y finalizaron 144 (79,1%). La prueba t para QOLIE-31 final entre grupos arrojó un t = −2,166 (IC [95%]: −10,125 -0,4625; p = 0,0319). Para el GI el cambio (Δ) en el QOLIE-31 fue de 12,45 puntos (p <0,001) y para el GC de 2,61 (p =0,072). Tomando 10,7 como cambio mínimo importante se encontró un Riesgo Relativo de 2,17 (IC: 1,37-3,43) y un Número Necesario a Tratar (NNT) de 3,5.

**Conclusiones:**

El estudio demostró que la aplicación del programa de AF mejora significativamente la CVRS en MCE. El NNT encontrado nos permite recomendar la implementación del programa de AF por el beneficio adicional que se obtendría en la CVRS de los pacientes.

## Introduction

Epilepsy is a complex neurological disorder that has deep physical, social, emotional and economic repercussions for patients and their environment. According to the World Health Organization, more than 20 million women worldwide suffer from epilepsy and 85% of them live in developing countries.

For the treatment of epilepsy, the gender of the patient should be considered because there are conditions, including hormonal (menstrual cycle, contraception and menopause), reproductive (fertility, pregnancy and lactation) and role (childcare) conditions, that can affect and be affected by anticonvulsant therapy and that change during the different stages of life of women. In women with epilepsy (WWE), major adverse reactions such as reproductive endocrine disorders, hirsutism, polycystic ovarian syndrome, obesity and sexual dysfunction have been reported, making the treatment of women more complex [1-3].

Several studies have shown that people with epilepsy have lower health-related quality of life (HRQOL), especially women [4-6], and have associated it with depression, adverse effects, a greater number of anticonvulsants and a higher frequency of seizures [7-10].

Anticonvulsants are considered the cornerstone of the treatment of epilepsy and are characterised by a narrow therapeutic index, high inter- and intraindividual variability, a large number of interactions and side effects and some of them have zero order kinetics.

It has been established that WWE require support and specific information in order to achieve adequate control of their condition. The European Declaration on Epilepsy recommends interdisciplinary action to help people with epilepsy understand their condition and make the search for a proper treatment possible in order to improve their quality of life [11,12].

Pharmaceutical care (PC) is the practice that optimises pharmaceutical treatments contributing to an improvement in HRQOL (humanistic outcome of therapeutic relevance) which can be reported directly by the patient as a patient-reported outcome (PRO) without requiring an interpretation of the response by others [13-16].

The pharmacist, as a professional healthcare expert on drugs, can contribute to the optimisation of treatment using medication review follow-up (MRF), education in pathology and lifestyle in the management of epilepsy, therapeutic drug monitoring (TDM) and information on interactions, adverse effects and the appropriate use of medications [17,18].

This clinical trial was designed with the aim of establishing the impact of the application of a pharmaceutical care programme on HRQOL in WWE.

## Patients and methods

The study was conducted in the Fundación Liga Central Contra la Epilepsia, sede Bogotá (LICCE), a non-profit organisation specialising in the treatment of outpatients with epilepsy and a referral centre for the management of this disorder in Colombia. Annually about 1000 patients consult for epilepsy, either referred by their health service or independently. An MRF service was implemented in the institution and a guide to medication review follow-up for patients with epilepsy was elaborated and used as the basis for the study intervention.

The study’s protocol was approved by the Ethics Committee of the LICCE on August 11, 2009 (ref: IEC-A1 Act 18 of 2009). All patients signed to give their informed consent before entering the study. The patients were invited to participate by posting notices at the institution and via phone calls to the WWE who met the inclusion criteria according to the review of the medical records of patients who were attending the LICCE. Patient recruitment began on June 16, 2010 and was completed on March 10, 2012. The latest follow-up interview took place on September 27, 2012.

### Study design

This study is a pragmatic, randomised controlled clinical trial (RCT) of parallel groups.

### Inclusion criteria

Women over 18 years of age were included if they had been diagnosed with epilepsy for over a year, were receiving out-patient treatment with anticonvulsants and had experienced at least one seizure in the last three years. Participants also had to have the ability to complete questionnaires.

### Exclusion criteria

WWE with psychiatric or neurological diseases diagnosed by a specialist that prevented them from making a judgement on their quality of life were excluded. Also excluded were patients with physical (e.g., hemiplegia) or mental deficits (e.g., mental retardation), making it impossible for them to answer the questionnaires, and those with a history of drug or alcohol abuse. Patients who had attended the LICCE’s MRF service before were also excluded.

### Intervention

A pharmaceutical care programme was applied, consisting of five parts:

1. Medication review follow-up according to Dáder’s method for a period of six months. Dáder’s method sought to discover drug-related problems (DRPs) in order to prevent and resolve drug-related negative outcomes (DNOs). This method is divided into seven stages – service offering, first interview, state of affairs, study phase, evaluation phase, intervention phase and subsequent interviews – rendering the process continuous [19]. In the service offering it was explained to the patient the kind of health care they would receive. In the first interview, information on health problems, use of medications and lifestyle habits were collected. In the state of affairs we established the relationship between DRPs and medications and in the study phase we searched for objective information about these. In the evaluation phase we identified the DNOs in order to establish needs and designed an intervention action plan for each patient. Subsequent interviews (monthly or bimonthly) were performed according to the conditions of each patient. If necessary, they were conducted by telephone.
2. Lectures given one Saturday a month in group education sessions on the following topics: Epilepsy in women, Quality of life and epilepsy, Pharmacological and non-pharmacological treatment in epilepsy, Contraception, Fertility, Pregnancy and childbirth, Sleep hygiene, Breastfeeding and homecare, Menopause and bone health and How to improve memory. These conferences were designed specifically for WWE and were given by the principal investigator in order to resolve questions and doubts. The dates of lectures were scheduled six months in advance and patients received a reminder e-mail or a call a few days before the conference.
  During the MRF interviews, education was reinforced in the different pathologies of each patient, in the proper use of medications, in different habits of life and in the management of adverse effects. A guide for patients with epilepsy [20] was sent by e-mail so that it could be discussed in subsequent interviews.
  In order to complement the verbal information, specific brochures were delivered according to the needs of each patient. These included: What to do in a seizure, Tips to improve sleep, Constipation management, Menopause management and bone health, Stress management, Effects of epilepsy on learning and memory, Breathing management, List of calcium-rich foods and Workbook for memory.
  At conferences and MRF interviews the importance of lifestyle was emphasised, including seven hours of continuous sleep, stress management and low consumption of alcohol or caffeine and other stimulants of the central nervous system.
3. Treatment adherence: the effect of anticonvulsants in controlling epilepsy was explained to patients, emphasising the importance of always taking them at the same time. For the patients who took multiple medications, a medication record was developed and aids were given to optimise adherence, such as a pill box (with a demonstration of its use) and an alarm clock as a reminder of when medications should be taken.
4. Registration of seizures and possible triggers: at the end of the first interview a seizure journal (calendar) with instructions on how to fill it out was given to patients in order to record the frequency of seizures and whether there were triggers (menstruation, sleep problems, stress, infection, fever or use of other drugs) that could be detected and used for the management of epilepsy.
5. Therapeutic drug monitoring of anticonvulsants was provided for patients who met any of the inclusion criteria in accordance with the guidelines of the International League Against Epilepsy [21]. They were given instructions for the proper conditions of the blood sampling and the cost was assumed by the study.

The PC programme was conducted by a pharmacist trained in the management of patients with epilepsy and the application of Dáder’s method [19].

### Control group

The patients in the control group (CG) received the usual care in the institution. The first interview was conducted according Dáder’s method for establishing baseline conditions, and a seizure journal (calendar) was given with instructions to fill it out for six months and then bring it to the second application of the questionnaires. These patients received the brochure “What to do in a seizure” with the respective explanation.

### Outcomes

The impact was assessed by the changes in seizure frequency, in the self-administered questionnaires (the Quality of Life in Epilepsy Inventory-31 (QOLIE-31), Liverpool Adverse Events Profile (Liverpool AEP), Center for Epidemiologic Studies Depression Scale (CES-D), Haynes-Sackett test and Moriski-Green test) and between the first interview and the one at the end of six months of follow-up. After each patient had answered the questionnaires, the pharmacist checked them and in case of missing answers, asked the patient to complete them.

The primary outcome was HRQOL measured by the QOLIE-31, translated into Spanish and validated in Spain’s version [22]. This questionnaire allows the quantification of the patient’s experience (PROs) by assessing seven areas of quality of life (Energy/Fatigue, Emotional well-being, Social functioning, Cognitive functioning, Medication effects, Seizure worry and Overall quality of life) with a Likert scale, allowing patients to obtain a score between 0 and 100 points, where a higher score indicates better quality of life. A QOLIE-31 overall score was derived by weighting and summing QOLIE-31 scale scores according the manual [16,23].

The following secondary outcomes were considered:

1. Frequency of crisis: a clinical variable for evaluating the effectiveness of treatment, it was classified into eight categories (see Table 1) and self-registered in a format by the patient [24].
2. Adverse reactions: determined with the Liverpool AEP translated and validated in Spanish. This 19-item questionnaire was designed to determine the frequency of the adverse effects of anticonvulsants and has the advantage of being short and easy to complete [16,25]. It rates between 19 and 76 and is inversely correlated with quality of life: scores below 45 are associated with low toxicity and those equal to or greater than 45 with high toxicity [26].
3. Depression: measured using the questionnaire of the CES-D, translated into Spanish and validated in Colombia [27]. This is one of the most used questionnaires to assess depression in a non-psychiatric population; it scores values between 0 and 60 where a higher score is associated with major depression [28]. In the validation in Colombia, a value of 20 was established as the cutoff point, since this value is considered to indicate that the patient has clinically significant depressive symptoms.
4. Adherence: at the end of the interview, the pharmacist administered the Haynes-Sackett test and the Moriski-Green test to assess patients’ adherence to drug treatment [29,30].

**Table 1:** **Sociodemographic and clinical characteristics**

**Characteristic**	**Initial patients No. (182)**	**Intervention group No. (70)**	**Control group No. (74)**	**Statistic**	**p-value**
Average age in years (SD)	34.2 (13.3)	34.7 (12.53)	36.2 (14.29)	0.6507	0.5163
Age range (years)	18 to 75	18 to 75	18 to 67		
Marital status - No. (%)				3.6093	0.3069
No partner	123 (67.6)	45 (64.3)	50 (67.6)		
Married or commonlaw	59 (32.4)	25 (35.7)	24 (32.4)		
Educational level - No. (%)				4.8222	0.438
Lower high school or below	32 (17.6)	10 (14.3)	17 (23.0)		
High school	110 (60.4)	43 (61.4)	40 (54.1)		
University	40 (22.0)	17 (24.3)	17 (23.0)		
Occupation - No. (%)				3.0313	0.6952
Working	78 (42.9)	31 (44.3)	32 (42.3)		
Housewife	33 (18.1)	11 (15.7)	15 (20.3)		
Student	40 (23.0)	17 (24.3)	13 (17.6)		
Retired	2 (1.1)	1 (1.4)	1 (1.4)		
Unemployed	29 (15.9)	10 (14.3)	13 (17.6)		
Epilepsy’s duration in years (SD)	16.8 (12.7)	17.9 (12.5)	17.2 (13.6)	−0.3078	0.7587
Age of onset in years (SD)	17.4 (12.5)	16.8 (11.8)	19.0 (14.2)	0.9602	0.3386
Epilepsy type (%)				0.3602	0.8352
Focal	95 (52.2)	38 (54.3)	39 (52.7)		
Generalised	79 (43.4)	29 (41.4)	33 (44.6)		
Indefinite	8 (4.4)	3 (4.3)	2 (2.7)		
Seizure frequency - No. (%)				5.9622	0.5442
One in the past 3 years	27 (14.8)	13 (18.6)	8 (10.8)		
One in the last year	36 (19.8)	17 (24.3)	15 (20.3)		
One every 6 months	21 (11.5)	5 (7.1)	11 (14.9)		
One every 3 months	25 (13.7)	8 (11.4)	9 (12.2)		
One per month	47 (25.8)	19 (27.1)	17 (23.0)		
One a week	12 (6.6)	5 (7.1)	7 (9.5)		
More than one a week but less than daily	10 (5.5)	2 (2.9)	6 (8.1)		
Daily	3 (1.6)	1 (1.4)	1 (1.4)		
Number of anticonvulsants - No. (%)				1.6932	0.1932
One	116 (63.7)	48 (68.6)	43 (58.1)		
More than one	66 (36.3)	22 (31.4)	31 (41.9)		

Other variables: in the first interview questions were included on occupation, marital status, education, family history of epilepsy, birth control and lifestyle habits, such as hours of continuous sleep, alcohol and coffee consumption and cigarette use.

Sample size was calculated using an “independent means comparison” method for the main outcome of quality of life. We expected a standard deviation of 12, mean differences from 50 to 55, a ratio between samples (B/A) of 1, a confidence level of 95% and a test power of 80%, whereby a sample size of 91 patients was obtained in each arm. To calculate the sample size an SD of 12 was used because in an Iranian study with epilepsy patients they reported that polymedicated patients had a QOLIE-31 of 46.98 (SD:12.07) [31], and in a preliminary study in the LICCE we found that 80.6% of WWE who attended the MRF service were being treated with polytherapy [32]. The Epidat 3.1 programme was used for calculations.

The random allocation sequence was generated by ballot papers drawn from an urn without the principal investigator and the coordinator knowing the results in advance. The allocation ratio was 1:1 for the two groups. The Runs test for randomness was applied to verify if the experimental units were allocated randomly to the two groups. The result (Z = −0 to 297, p-value =0.7662) shows that there is no statistical evidence to reject the hypothesis of complete randomness in the order of allocation of the patients.

The concealment was performed by placing the ballot papers in individual, opaque, sealed envelopes, numbered sequentially, which were handled exclusively by the study coordinator.

Patients were recruited by the pharmaceutical programme officer after scrutiny of the inclusion–exclusion criteria and the acquisition of signed informed consent. Immediately after the admission of a patient to the study, the coordinator proceeded to open the envelope numbered sequentially to establish the group that she entered and register it. The patients did not receive money for their participation in the study but they were reciprocated with aids to improve treatment such as brochures, pill boxes, alarm clocks, top-eyes, top-ears, an elaboration of “Registering taking medication” and free TDM of anticonvulsants.

Although the study was not blinded, it was explained to the patients that due to the large number of patients, all could not be served at the same time and therefore the study was conducted in two stages whose sequence was decided randomly, so they could begin the process of PC immediately, or do it six months after the second questionnaire session. In this way the effect of knowing the group assigned was avoided and those in the CG were rewarded for their participation in the study programme by receiving PC after answering the questionnaires the second time.

The study was blind to the LICCE neurologists. They were informed that the RCT was taking place in the institution but did not know which patients were participating in the trial. In the MRF service we attended LICCE patients who were not included in the RCT. Due to the study’s design, the principal investigator was not blinded to the patients’ allocation.

Statistical methods: Through the evaluation of the study results, the null hypothesis of the equality of mean scores in the QOLIE-31 after application of the PC programme was established (μIG = μCG) versus the alternative, that mean scores are different (μIG ≠ μCG).

A complete case analysis was conducted, omitting the data from lost patients. It was considered inappropriate to perform data analysis by imputation because for the second HRQOL assessment the change (Δ) in each patient needed to be established.

An analysis for intention to treat was done with the patients who completed the second application of questionnaires, although these patients had not attended the MRF interviews or the health education lectures in the PC programme.

An exploratory and descriptive data analysis was conducted for the sociodemographic and clinical characterisation of the patients via the information registered in interviews and medical records. In addition, it was verified using a Student’s t-test and a Chi-square test that there were no significant differences between groups at baseline. The two-sample Kolmogorov-Smirnov test was used to compare the cumulative distributions of the initial scores of the QOLIE-31.

To quantify the effect of the PC programme on HRQOL, the means of the scores were compared between groups at the end of the study with an independent samples Student’s t-test. The change (Δ) in scores (QOLIE-31 after-QOLIE-31 before) in the two groups was evaluated using a paired Student’s t-test. The assumptions of normality in the residuals and homogeneity of variances between groups were checked with the Shapiro-Wilk test and Bartlett’s test respectively. Data processing was performed in the R programme (version 3.0.1) [33].

To evaluate the clinical significance of the application of the PC programme, an increase of 10.7 points in the QOLIE-31 was established as the minimally important change (MIC) according to studies by Cramer et al. and Borghs et al. [34,35]. To estimate the strength of association the relative risk (RR) and number needed to treat (NNT) were calculated. Finally, the calculation of RR for the best- and the worst-case scenario was performed.

## Results

**Figure 1:**
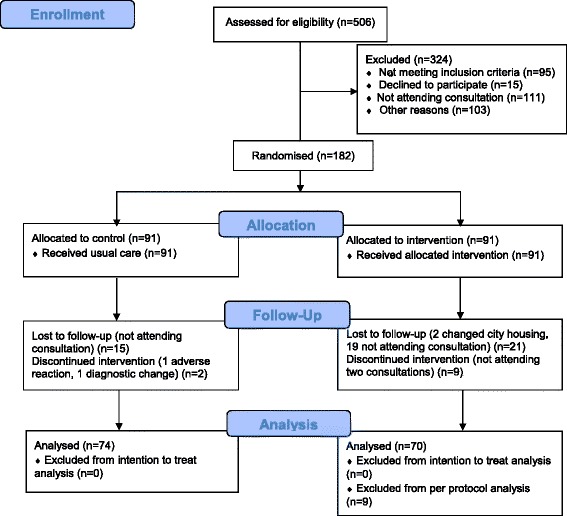
**Flow diagramme.**

We invited 506 patients to participate, of whom 182 entered the study and 144 (79.1%) completed it, 70 in IG and 74 in CG. The Reasons for withdrawal were job, economic and family-related and in no case related to the outcome (Figure 1).

The most widely used anticonvulsant was valproic acid in 85 patients (46.7%), while carbamazepine was used in 42 (23%), levetiracetam in 22 (12.1%) and lamotrigine in 20 (11%). Most patients (116) were on monotherapy (63.7%), which is highly recommended for the management of this condition, and only four (2.7%) received three anticonvulsants.

The average value of the QOLIE-31 for the initial group of 182 patients was 54.56, and confidence interval (CI 95%) 53.79; 55,33. The two-sample Kolmogorov-Smirnov test between scores of the initial groups showed a p-value =0.1686. The homogeneity variances test of the initial scores of the groups resulted in F =1.02 and a p-value of 0.94. The t-test results relating to the difference of the initial mean scores between groups were t = 0.8499, and CI 95%: −2.56; 6.44, p-value: 0.397.

The t-test of the final mean scores of the QOLIE-31 between groups was t = −2.166, CI 95%: −10.125; −0.4625, p-value =0.0319.

The mean of the change (Δ) (after-before) in the QOLIE-31 scores in the final group of 144 patients was 12.45 points in the IG and 2.61 points for the CG. In the paired t-tests comparing initial and final scores, the following results were obtained: for the IG t =8.1878 (CI: 9.41; 15.48, p-value <0.001) and for the CG t =1.8259 (CI: −0.24; 5.45, p-value =0.072).

**Figure 2:**
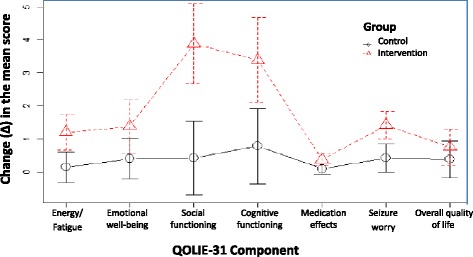
**Changes (after-before) in the Quality of Life in Epilepsy Inventory-31 (QOLIE-31) scores by components.**

**Figure 3:**
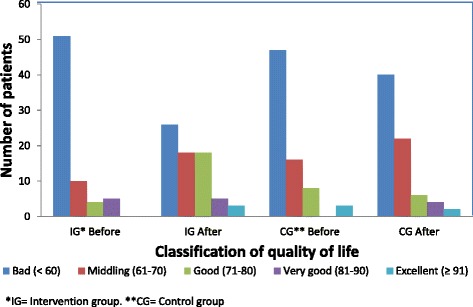
**Classification of quality of life according to the initial and final QOLIE-31 scores.**

An analysis was performed for each component of the QOLIE-31 to establish the effect of the PC programme. The changes (Δ) (after-before) indicated by the scores for each of the seven components of the QOLIE-31 are presented in Figure 2. Categorisation of the QOLIE-31 scores before and after application of the PC programme was performed according to quality of life (see Figure 3) [36].

The mean of the change (Δ) in the QOLIE-31 scores for IG patients who attended at least three MRF interviews (61/70) during the application of the PC programme (analysis per protocol) was 14.41 (CI: 11.37; 17.45).

The contingency table was made with an increase of 10.7 points in the QOLIE-31 score taken as the minimally important change [34]. Epidemiological parameters were calculated finding a RR of 2.17 (CI: 1.37; 3.43) and a NNT of 3.5. The RR was 3.22 for the best-case scenario and 1.06 for the worst-case scenario.

Attendance at health education conferences fluctuated between 28% and 3%. The seizure journal was returned six months after, at the end of the study, by 54 (37.5%) of the 144 patients.

The results of the Liverpool AEP, CES-D, Haynes- Sackett and Moriski– Green tests will be published in subsequent papers.

## Discussion

To our knowledge this is the first RCT investigating the impact of a programme of PC on HRQOL among patients with epilepsy.

The number of patients lost to follow-up was similar in both groups. Table 1 reveals that the characteristics of the groups, after losses, do not differ from those of the entire group of patients who entered the study. The initial QOLIE-31’s mean scores, of the patients who remained in the study, did not present statistically significant differences between groups (Table 2).

**Table 2:** **The quality of life in epilepsy inventory-31 scores**

**Quality of life questionnaires**	**Intervention group No (70)**	**Control group No (74)**
QOLIE-31 before (CI 95%)	51.9 (50.40; 56.78)	56.5 (52.31; 58.74)
QOLIE-31 after (CI 95%)	64.4 (61:00; 67.78)	59.1 (55.61; 62.58)
Change (Δ) QOLIE-31 after- before	12.45 (9.41; 15.48)	2.61 (−0.24; 5.45)
p-value	<0.001	0.072

The t-test for the final scores of the QOLIE-31 established a statistically significant difference (p-value =0.0319) with an increase in the HRQOL after the application of the PC programme in the IG. The paired t-tests to compare the initial and final scores showed that there is a statistically significant difference in the change of the scores of the QOLIE-31 in the IG (p-value <0,001), but not in the CG (p-value =0,072) confirming that the intervention does serve to improve HRQOL in WWE.

The increase of 12.45 points in the QOLIE-31 scores of the IG is considered clinically significant according to the study by Borghs et al. and Cramer et al. of the Epilepsy Impact Project Group, where they established the minimally important change of 10.7 points for the QOLIE-31 in patients passing to monotherapy, who are the most similar to the patients in this study [34,35].

The individual analyses of the seven components of the QOLIE-31 allowed us to establish that the changes (Δ) were positive in all cases and they were always higher for the IG (Figure 2). Seizure worry, social and cognitive functioning presented significant differences. It is possible that the improvements in social and cognitive functions are due to the clarifications made in the MRF interviews, to the WWE and his family, about the use of medications, diseases, lifestyle habits (allowed to have up to 2 beers/celebration, attend evening meetings, adjusting sleeping schedules to 7 h uninterrupted, the importance of hobbies and activities for stress management, etc.). Learning this information from a pharmacist and resolving their doubts could have led to a reduction in their fear of social activities and the self-stress of living with this disease. The increase in HRQOL in the drug component was small, possibly due to the fact that the follow-up period of six months is too short for evaluating the benefits of optimized drug therapy. Additionally, the QOLIE-31 includes only three questions to evaluate the effect of drugs and gives them the lowest weight (0.03) throughout the questionnaire, while a total of eleven questions are devoted to either social or cognitive function and they are given the greatest weight in the questionnaire (0.21 and 0.27 respectively) [23].

Most patients had bad HRQOL at baseline (Figure 3), which remained in the CG at the end of the study (p-value =0.1994), but decreased almost to half in the IG, presenting a statistically significant difference (p-value <0.001) which makes the positive effect of the PC programme on HRQOL among WWE evident [37].

Helde et al. conducted a RCT to evaluate the change in HRQOL with a two-year application of a structured nurse-led programme among patients with epilepsy [38]. They used the QOLIE-89, which is similar to the QOLIE-31, and found an initial value of 52.5 ± 1.3 (standardised error margin) and two years later a change (Δ) of 2.3 ± 7.0 in the IG and 1.5 ± 7.2 for the CG, which is not clinically significant, according to Wiebe et al., who established an MIC of 10.1 points for the QOLIE-89 [39].

In the per protocol analysis, an even greater increase in quality of life for IG patients who attended at least three interviews of the PC programme during the six months (Δ) was observed: 14.41 points (CI: 11.37; 17.45). This result highlights the importance of the continuity of the process.

The average value of the QOLIE-31 for the initial group of 182 patients was 54.56 ± 15.36 (SD), similar to the one found by Almeida Souza Tedrus et al. with a value of 58.37 ± 17.31 (SD) for WWE in Brazil [4], where the gap between women and men was statistically significant at 7.57 points (p-value: 0.012). It was also similar to the value of 53.4 ± 14.7 (SD) reported by Alanis-Guevara et al. for WWE in Mexico [5]. In both cases HRQOL was lower in women [6].

In a prospective study performed by Kanjanasilp et al., PC was provided for six months among patients with epilepsy treated with phenytoin; they measured HRQOL before and after the intervention with the QOLIE-31 and found that it increased from 61.15 ± 13.67 to 63.47 ± 16.11, showing a statistically significant increase of 2.32 points [40].

The increase in the QOLIE-31 scores in our CG in the second application could be due to the fact that during the first interview, according to Dáder’s method, patients expressed their doubts regarding treatment and pathologies, and the pharmacist, for ethical reasons, had to answer the concerns and correct the patients when errors were found in the administration of medications, although this could lead to the contamination of the group. In a before-after study conducted in adults with epilepsy by Fogg et al. [41], the effect of a single 30-minute consultation with a pharmacist was evaluated using the QOLIE-10, and they found that HRQOL increased by 4.2 points (CI 95%: 0.4; 8.0). Although this is a different questionnaire, an increase in HRQOL due to a single consultation with a pharmacist was observed, just as it was in our CG.

The clinical significance of the implementation of the PC programme was evaluated (see Table 3), finding that the possibility of obtaining an MIC in the IG is 2.17 times the CG’s one, even though that PC was presented to the CG during the first interview. The result could have been higher if this confounding bias had not been introduced.

**Table 3:** **Contingency table for the implementation of the pharmaceutical care programme**

**Exposure to the programme**	**Increase (Δ) ≥10.7**	**Increase (Δ) <10.7**	**Total**
Intervention group	37	33	70
Control group	18	56	74
Total	55	89	144

By performing the sensitivity analysis, we found that for both the best- and the worst-case scenario the value of RR is higher than 1, confirming that the intervention does serve to improve HRQOL and that the result is due to the implementation of the PC programme. Sensitivity analysis also allows us to assess the impact of loss of follow-up. We found the values were not very different from each other (RR for the worst scenario: 1.06 and for the best scenario: 3.22). We deduce from this that the losses did not affect the validity of the study. Additionally, we believe that the results were not biased because the losses were not related to HRQOL. The effect of the intervention is notable because, despite the losses, which led to a significant reduction in sample size (and in the power of the study), clinically and statistically significant differences were found in both the ITT analysis and in the PP analysis.

The NNT found allows us to recommend the implementation of the PC programme for the additional benefit that would be obtained in patients’ HRQOL.

Unfortunately, conference attendance was very low despite being scheduled six months in advance, with the most common reason for the absence being lack of time.

The seizure journal was delivered by only 54 (37.5%) of the 144 patients, possibly because they had never used it and therefore did not realise its importance, despite the explanation and recommendation about filling it out. This result could also be due to the fact that it was given at the end of the first interview, after about an hour of consultation with the application of five questionnaires, and patients were tired and may not have understood the purpose of completing this seizure journal.

We believe that the study by a single researcher, qualified and experienced in the management of patients with epilepsy and in the implementation of pharmaceutical care, allowed an homogeneous intervention. The bias of the researcher was minimised by measuring the outcomes with a patient-reported outcome questionnaire that does not require interpretation.

Initially, an inclusion criterion of having had at least one crisis in the last year (85.2% met this condition) was established, focussing on patients with greater difficulty controlling the disease, but given the slow rate of recruitment, the decision was made to extend this criterion to at least one seizure in the last three years.

### Study’s limitations

Due to the study design, the pharmacist who performed the procedure was not blinded to the allocation of the patients.

Of the QOLIE-31 versions available in Spanish, Spain’s version was selected for being very similar to Colombia’s Spanish. According to our knowledge, of all the questionnaires used, only the CES-D is validated in Colombia.

## Conclusions

The study allowed us to demonstrate that the application of a pharmaceutical care programme significantly improves HRQOL in WWE. The NNT we found allows the recommendation to implement the PC programme for the additional benefit that would be obtained in patients’ HRQOL.

### Recommendations

Institutions serving WWE should implement pharmaceutical care programs as a public health measure to improve the HRQOL.

It is recommended to include the pharmacist in the health team serving the WWE.

It is not convenient to apply five questionnaires in one sitting because it is very tedious for the patients and it may induce withdrawal from the study.

## Abbreviations

CES-D: Spanish Center for Epidemiologic Studies Depression Scale
DNOs: Drug-related negative outcomes
DRPs: Drug-related problems
HRQOL: Health-related quality of life
LICCE: Fundación Liga Central Contra la Epilepsia, sede Bogotá
Liverpool AEP: Liverpool Adverse Events Profile
MIC: Minimally important change
MRF: Medication review follow-up
NNT: Number needed to treat
PC: Pharmaceutical care
PRO: Patient-reported outcome
QOLIE-10: Quality of Life in Epilepsy Inventory-10
QOLIE-31: Quality of Life in Epilepsy Inventory-31
QOLIE-89: Quality of Life in Epilepsy Inventory-89
RCT: Randomised controlled trial
RR: Relative risk
TDM: Therapeutic drug monitoring
WWE: Women with epilepsy
